# Oyaksungisan, a Traditional Herbal Formula, Inhibits Cell Proliferation by Induction of Autophagy *via* JNK Activation in Human Colon Cancer Cells

**DOI:** 10.1155/2013/231874

**Published:** 2013-03-14

**Authors:** Nam-Hui Yim, Young Pil Jung, Aeyung Kim, Choong Je Ma, Won-Kyung Cho, Jin Yeul Ma

**Affiliations:** ^1^Korean Medicine- (KM-)Based Herbal Drug Research Group, Korea Institute of Oriental Medicine (KIOM), Daejeon 305-811, Republic of Korea; ^2^Division of Bioscience and Biotechnology, Department of Biomaterials Engineering, Kangwon National University, Chuncheon 200-701, Republic of Korea

## Abstract

Oyaksungisan (OY) is a traditional herbal formula broadly used to treat beriberi, vomiting, diarrhea, and circulatory disturbance in Asian countries from ancient times. The effect of OY on cancer, however, was not reported until now. In this study, we have demonstrated that OY inhibits cell proliferation and induces cell death *via* modulating the autophagy on human colon cancer cells. In HCT116 cells, OY increased the ratio of LC3-II/LC3-I, a marker of autophagy, and treatment with 3-MA, an inhibitor of autophagy, and considerably reduced the formation of autophagosomes. In addition, OY regulated mitogen-activated protein kinase (MAPK) cascades; especially, JNK activation was closely related with autophagy effect by OY in HCT116 cells. Our results indicate that autophagy induction is responsible for the antiproliferative effect by OY, despite the weak apoptosis induction in HCT116 cells. In conclusion, OY might have a potential to be developed as an herbal anticancer remedy.

## 1. Introduction

Autophagy is a self-protective cellular mechanism providing energy through the degradation and recycling of cytoplasmic constituents [1]. Autophagic cells are well characterized by the accumulation of vacuoles at the beginning of autophagy and sequestration of cytoplasmic portion in double-membrane bound which are known as autophagosomes [2]. Autophagy is involved in many aspects of health and development, including aging, pathogenic infection, stress responses, neurodegenerative and muscle disorders, and cellular remodeling [3, 4]. Since rapidly proliferating cancer cells need nutrient supply, cancer cells are likely to use autophagy to obtain ammonia acids as alternative energy sources [5]. By contrast, most cancer cells including colon, breast, prostate, and brain undergo autophagic cell death after anti-cancer therapies [6].

Advanced cancer is a multifactorial disease that demands treatments targeting multiple cellular pathways. In addition, drug toxicity and resistance on chemotherapeutic agents make a struggle to treat cancer. For these reasons, nontoxic dietary phytotherapy has been considered as a preventative and/or therapeutic method against cancer cells [7]. Traditional oriental herbal medicines have been used for treatment of malignant cancers. Among them, a number of herbal cocktails have been reported to have antitumor activities and some of them have been used by cancer patients for a long time [8–13]. Herbal cocktail consisting of various constituent herbs could affect multiple cellular pathways, thereby modulating cellular functions formed during cancer development. It is believed that a herbal cocktail formulated properly takes advantage of synergy effect, and interactions of phytochemicals present in the different herbs may achieve better therapeutic efficacy than single herbs [14]. 

Oyaksungisan (OY) is a traditional herbal medication, broadly used in Asian countries and has been prescribed to treat beriberi, vomiting, diarrhea, and circulatory disturbance for several decades [15]. Recently, numerous studies have reported the bioactivities of OY, such as neuroprotection [16], anti-H_2_O_2_-induced apoptosis [17], and anti-inflammation effect [15]. OY is an aqueous polyherbal formulation and consists of twelve herbs: Ephedra Herb, Citrus Unshiu Peel, Lindera Root, Cnidii Rhizoma, Angelica Dahurica Root, Batryticatus Bombyx, Aurantii Fructus Immaturus, Platycodon Root, Zingiberis Rhizoma, Glycyrrhizae Radix et Rhizoma, Zingiberis Rhizoma Crudus, and Zizyphi Fructus. Although some single herbs in OY, including Citrus Unshiu Peel [18], Lindera Root [19], Angelica Dahurica Root [20], and Zingiberis Rhizoma [21] were reported to have an inhibitory activity against cancer, anti-cancer effect of OY is still not investigated. In this study, we first demonstrate that anti-cancer effect of OY is arisen from synergistic effect of constituent herbs and is related with autophagy induction in human colon cancer cells. 

## 2. Materials and Methods

### 2.1. Chemicals and Reagents

For analyzing the main components of herbs in OY, ferulic acid was purchased from Sigma-Aldrich (USA). Ephedrine-HCL, 6-gingerol, glycyrrhizin, imperatorin, and hesperidin were purchased from Korea Food & Drug Administration (KFDA). HPLC grade solutions (water and acetonitrile) were purchased from J. T. Baker Chemical Co. (Pillipsburg, NJ, USA). DMEM and RPMI-1640 mediums for cell culture were purchased from Lonza (Wakersville, MD, USA). Penicillin G/streptomycin and Trypsin/EDTA were obtained from Gibco (Grand Island, NY, USA). Fetal bovine serum (FBS) and phosphate-buffered saline (PBS) were obtained from Hyclone (Logan, UT, USA) and WElGENE (Daegu, Republic of Korea), respectively. Dimethyl sulfoxide (DMSO), 3-[4,5-dimethylthiazol-2-ly]-2, 5-diphenyltetrazolium bromide (MTT), 3-methyladenin (3-MA), and anti-LC3 antibody was purchased from Sigma-Aldrich (St. Louis, MO, USA). Protease and phosphatase inhibitors cocktail were purchased from Roche Diagnostics (Mannheim, Germany). RIPA buffer was obtained from Millipore (Billerica, MA, USA). Cytotoxicity detection kit (lactate dehydrogenase, LDH) was purchased from Roche Diagnostics (Mannheim, Germany). Primary antibodies against p38, p-p38, ERK, p-ERK, JNK, p-JNK, Bcl-2, cytochrome c (Cyt. c), caspase-3, -9, PARP, and secondary antibodies were purchased from Cell Signaling (Danver, MA, USA) and *β*-actin was purchased from Santa Cruz Biotechnology (Santa Cruz, CA, USA). 

### 2.2. Herb Materials and Preparation of OY

OY is composed of twelve herbs and their constitution ratio is listed in Table 1. Twelve medicinal herbs of OY were purchased from the Korea Medicine Herbs Association (Yeongcheon, Republic of Korea). The mixture of medicinal herbs was extracted by heating in water of 8–10 times of herb weight for 3 h at 115°C (Gyeongseo Extractor Cosmos-600, Incheon, Republic of Korea). After boiling, the extract was filtered out using standard testing sieves (150 *μ*m) (Retsch, Haan, Germany) and prepared in the form of powder by freeze drying. Fifty mg of OY powder were dissolved in 1 mL of PBS and stored at −20°C before use.

### 2.3. Chromatographic Conditions

The standard compounds (ephedrine-HCl, ferulic acid, hesperidin, 6-gingerol, imperaton, and glycyrrhizin) and the powder of OY were accurately weighed and dissolved in 60% methanol. Those were stored at 4°C and filtered through a 0.45 *μ*m membrane filter before HPLC analysis. The high performance liquid chromatography-diode array detector (HPLC-DAD) system (Dionex, USA) consisted of a pump (LPG 3X00), autosampler (ACC-3000), column oven (TCC-3000SD), and diode array UV/VIS detector (DAD-3000RS). System control and data analysis were performed using Dionex software. The analysis of OY and standard compounds was conducted using a LUNA C_18_ column (5 *μ*m, 4.6 mm × 250 mm). The mobile phase consisted of water with 0.1% Trifluoroacetic acid (TFA) (A) and acetonitrile (B) at a flow rate of 1.0 mL/min and the column temperature was maintained at 35°C. The applied elution conditions were laid out in Table 2. The standard compounds and components of OY were detected at suitable UV wavelengths such as 207, 250, 280, and 320 nm. 

### 2.4. Cell Culture

Several human cancer cells were obtained from the Korean Cell Line Bank (KCLB, Seoul, Republic of Korea) and American Type Culture Collection (ATCC, Rockville, MD, USA). These cells were cultured in DMEM or RPMI-1640 with 10% FBS, and primary hepatic cells isolated from Imprinting Control Region (ICR) mice were grown in Williams Medium E (Gibco, USA) supplemented with 10% FBS. All media contained 100 unit/mL Penicillin G and 100 *μ*g/mL streptomycin. All cells were cultured in an atmosphere of 5% CO_2_ at 37°C. 

### 2.5. Cell Viability Assay

Cell viability was determined using MTT colorimetric assay, based on the reduction of tetrazolium salt to its insoluble formazan. The cells were seeded in a 96-well culture plate (4 × 10^3^ or 5 × 10^3^ cells/well) and treated with OY for 24 or 48 h. After incubation, 10 *μ*L of MTT working solution (5 mg/mL in PBS) were added to each well and further incubated at 37°C for 4 h. After removing the media, formazan precipitates were dissolved with 100 *μ*L DMSO. Absorbance at 570 nm was measured using a microplate reader (Sunrise, TECAN, Männedorf, Switzerland) and the cell viability was determined as a percentage of viable cells compared with untreated, control cells. To determin cytotoxicity induced by OY, LDH released from HCT116 cells was evaluated with the commercial kit according to the manufacturer's instructions.

### 2.6. Western Blot Analysis

The cells treated with OY were washed twice with cold PBS and lysed in RIPA buffer containing protease and phosphatase inhibitors cocktail for 30 min on ice. The lysates were centrifuged at 15,000 ×g for 20 min at 4°C and supernatant was used for western blot analysis. The same amount of protein for each sample was electrophoresed and transferred onto polyvinylidene difluoride (PVDF) membrane (Pall Bio support Division, Port Washington, NY, USA). After blocking the membranes in Tris-Buffer saline containing 5% (w/v) skim milk and 0.1% Tween 20 for 1 h, the membranes were incubated with a primary antibody (1 : 1000) at 4°C overnight and followed by incubation with the corresponding horseradish peroxidase-conjugated secondary antibody (1 : 5000) at 37°C for 1 h. The specific protein was detected with ECL solution (Thermo Fisher Scientific, Rockford, IL, USA) using the Davinch-chemi Chemiluminescence Imaging System CAS-400SM (CoreBio, Seoul, Republic of Korea).

### 2.7. Detection of Autophagy

The induction of autophagy was evaluated by checking the formation of autophagosome into the cells and detecting an increase of microtubule-associated protein light chain 3 (LC3). Cells were treated with OY for 24 h with or without pretreatment of 10 mM of 3-MA for 1 h. Cell morphology was observed under a phase-contrast microscope (OLYMPUS, JAPAN), and cell viability was determined using MTT assay. The conversion of LC3-I to LC3-II by OY treatment was detected with Western blot analysis.

### 2.8. Statistical Analysis

Data were presented as mean ± SD. Student's *t*-test was employed to assess the statistical significance between control and OY-treated cells. A *P* value less than 0.05 was considered as statistically significant.

## 3. Results 

### 3.1. Representative Chromatograms of Several Constituents in OY

**Figure 1:**
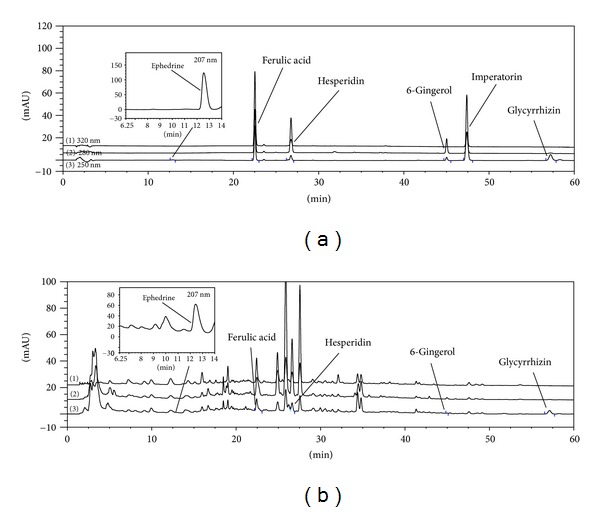
HPLC fingerprints of OY. (a) HPLC profiling of standard components of constituent herbs contained in OY. (b) Identification of components in OY prescription by HPLC. HPLC chromatograms of components were combined after monitoring at 250, 280, and 320 nm, respectively. Ephedrine, ferulic acid, hesperidin, 6-gingerol, imperatorin, and glycyrrhizin were determined as constituents of Ephedra Herb, Cnidii Rhizoma, Aurantii Fructus, Zingiberis Rhizoma, Angelica Dahurica Root, and glycyrrhizin from Glycyrrhizae Radix, respectively. The retention times of standards for the constituent herbs of OY were detected at *t*_*R*_ 12.86, 22.52, 26.75, 45.02, 47.39, and 57.22 min. Five components in OY were detected at *t*_*R*_ 12.76, 22.43, 26.62, 44.96, and 57.13 min.

The constituents of OY were determined by HPLC analysis and each peak of UV spectra was compared with that of representative standard compounds. As described in Figure 1(a), HPLC-DAD analysis revealed that single representative peaks of each chemical standard of component herbs contained in OY appeared at various retention times. UV spectrum analysis of reference compounds identified the following known constituents of OY: ephedrine-HCl from Ephedra Herb, ferulic acid from Cnidii Rhizoma, hesperidin from Aurantii Fructus, 6-gingerol from Zingiberis Rhizoma, and glycyrrhizin from Glycyrrhizae Radix. Imperatorin from Angelica Dahurica Root was not detected in this analysis.

### 3.2. OY Reduces Cell Viability on Human Colon Cancer Cells

**Figure 2:**
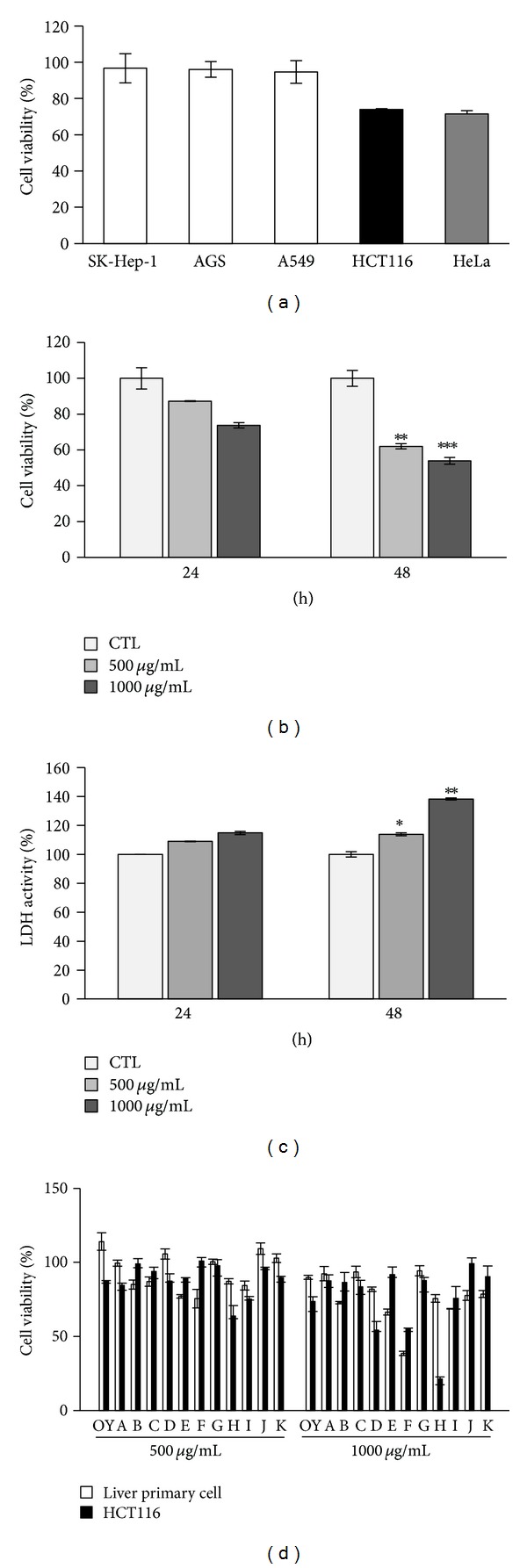
Inhibitory effect of OY on the cell proliferation in human cancer cells. (a) Inhibition of cell viability by OY in several human cancer cells. Liver (SK-Hep-1), stomach (AGS), lung (A549), colon (HCT116), and cervical (HeLa) cancer cells were treated with OY (500 *μ*g/mL) and incubated for 48 h. Cell viabilities were determined by MTT assay. (b) Dose- and time-dependent effect of OY on the viability of HCT116 cells. Cells were treated with 500 and 1000 *μ*g/mL for indicated time, and viabilities were determined using MTT assay. Each data value indicates the mean ± SD. **P < 0.01 and ***P < 0.001 versus untreated cells. (c) Dose- and time-dependent LDH release by OY in HCT116 cells. Cells were treated with 500 and 1000 *μ*g/mL for indicated time, and the release of LDH was determined using LDH detection kit. Each data value indicates the mean ± SD. *P < 0.05, **P < 0.01, and ***P < 0.001 versus untreated control cells (CTL). (d) Comparison of cytotoxicity between OY and its constituent herbs on cancer cells and mouse liver primary cells as a normal cells, respectively. Cells were treated with OY or each constituent herbs contained in OY at concentrations of 500 *μ*g/mL and 1000 *μ*g/mL for 24 h, respectively. Cell viabilities were determined using MTT assay. The results were expressed as a percentage of viable cells compared to untreated control cells. A, Cnidii Rhizoma; B, Angelica Dahurica Root; C, Glycyrrhizae Radix et Rhizoma; D, Citrus Unshiu Peel; E, Batryticatus Bombyx; F, Platycodon Root; G, Aurantii Fructus Immaturus; H, Ephedra Herb; I, Zingiberis Rhizoma; J, Lindera Root; K, Zizyphi Fructus.

The anti-proliferative effect of OY on several human cancer cells was examined using MTT assay. OY was treated with 500 *μ*g/mL for 48 h on five kinds of human cancer cell lines including SK-Hep-1 (liver), AGS (stomach), A549 (lung), HCT116 (colon), and HeLa (cervical) cells. The cell proliferation of HCT116 and HeLa cells was, respectively, inhibited up to 27% and 29% by OY treatment, while three cancer cell lines, such as SK-Hep-1, AGS, and A549, were not affected by OY (Figure 2(a)). In this study, we focused on anti-cancer effect on HCT116 cells. To more define the anti-cancer effect of OY on colon cancer cells, we checked dose- and time-dependent cell death effect by OY. As shown in Figure 2(b), after treatment with OY for 24 h, cell viability of HCT116 cells was reduced up to 13% and 26% at 500 *μ*g/mL and 1000 *μ*g/mL, respectively. In case of long-term treatment with OY (48 h), viability was significantly reduced to 38% and 46% at 500 *μ*g/mL and 1000 *μ*g/mL, respectively, which was about 2-fold increase compared to that at 24 h. In Figure 2(c), LDH release was also increased according to the concentration and incubation time of OY treatment in HCT116 cells. In contrast, at 24 h post-treatment, the viability of mouse liver primary cells used as normal control cells was not affected by OY at the same concentrations exhibiting the cytotoxicity on HCT116 cells. On the basis of these results, inhibition of cell viability by each medicinal herbs contained in OY was examined at the same concentration used for OY in HCT116 and normal cells. Most herbs at concentration of 1000 *μ*g/mL showed weak anti-proliferative effects except for Citrus Unshiu Peel (D), Platycodon Root (F), Ephedra Herb (H), or Zingiberis Rhizoma (I) on HCT116 cells (Figure 2(d)). These four component herbs of OY exhibited much higher anti-proliferative effect against HCT116 cells than that of OY at 1000 *μ*g/mL concentration and inhibited the proliferation of mouse liver primary cells up to 19% (D), 61.3% (F), 24.3% (H), and 31.3% (I), which showed stronger cytotoxicity than that of OY at the same concentration. Angelica Dahurica Root (B) and Batryticatus Bombyx (E) exhibited strong cytotoxicity on normal cells without anti-cancer effects (Figure 2(d)). These results indicate that OY contains a specific anti-cancer effect on colon cancer cells through covering the toxicity of twelve medicinal herbs.

### 3.3. OY Mediates Autophagic Molecular Events in HCT116 Cells

**Figure 3:**
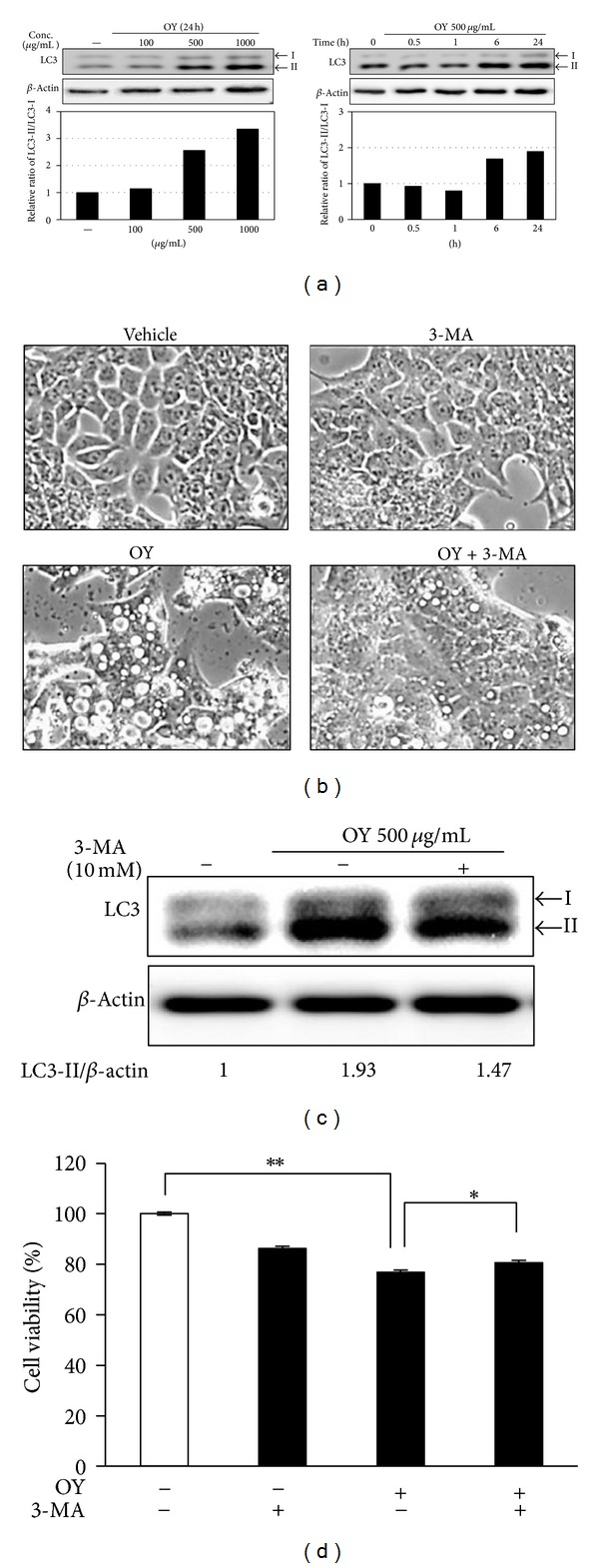
Induction of autophagy by OY in HCT116 cells. (a) Cells were treated with indicated doses of OY for 24 h or treated with 500 *μ*g/mL OY for indicated times before lysis. Western blot analyses were performed for checking the protein levels of LC3-I and -II. The band intensities were quantified by Image J program and are represented in the bar graph. *β*-Actin was used as a loading control for normalization. (b) To confirm the autophagy induction by OY, cells were treated with 1000 *μ*g/mL OY for 24 h with or without pretreatment with 10 mM 3-MA for 1 h. After 24 h of OY exposure and 3-MA, cell morphology was observed under a phase contrast microscope. (c) Cells were treated with 500 *μ*g/mL OY with or without preexisting 10 mM 3-MA for 3 h, and the upregulation of LC3-II level by OY was determined as a Western blot analysis. The band intensities were quantified by Image J program, and values indicate relative levels determined after normalization to untreated cells. (d) To confirm the autophagic effect of OY against cell proliferation on HCT116 cells, cells were treated with 1000 *μ*g/mL OY with or without pre-existing 10 mM 3-MA for 24 h. After 24 h of OY exposure and 3-MA, cell viability was determined using MTT assay. Values indicate the mean ± SD. **P < 0.01 versus untreated control cells. *P < 0.05 versus treated cells with OY only.

Autophagy is generated by the accumulation of autophagosomes in cells, which is estimated by detecting the level of LC3 [22]. It is well known that LC3-II/-I ratio directly correlates with the formation of autophagosomes [23]. To determine the induction of autophagy by OY in HCT116 cells, the cells were treated with various concentrations of OY for various time points. At first, we analyzed the extent of the conversion of LC3-I into LC3-II using Western blotting. As shown in Figure 3(a), the ratio of LC3-II to LC3-I was increased by treatment with OY at the concentrations of 500 *μ*g/mL and 1000 *μ*g/mL for 24 h compared to untreated control. Also, the cleavage of LC3-II from LC3-I was remarkably elevated from 6 h of 500 *μ*g/mL OY exposure. In Figure 3(b), autophagy induction by OY in HCT116 cells was confirmed using 3-MA, a pharmacological autophagy inhibitor. We have observed that OY at 1000 *μ*g/mL causes the accumulation of big cytoplasmic vacuoles in HCT116 cells, while pretreatment with 10 mM 3-MA reduced vacuole formation in the cells. In addition, the increase of LC3-II level by OY was considerably decreased by pre-treatment with 3-MA, suggesting that 3-MA blocks autophagy induction by OY (Figure 3(c)). However, treatment with 3MA did not completely recover the cell viability reduced by OY in HCT116 because 3-MA treatment itself showed somewhat cytotoxicity (Figure 3(d)). These results strongly suggest that OY induces autophagy through the regulation of LC3-II/-I protein levels and enhances the cell death of HCT116 cells. 

### 3.4. OY Induces Autophagy Process *via* JNK Activation in Human Colon Cancer Cells

**Figure 4:**
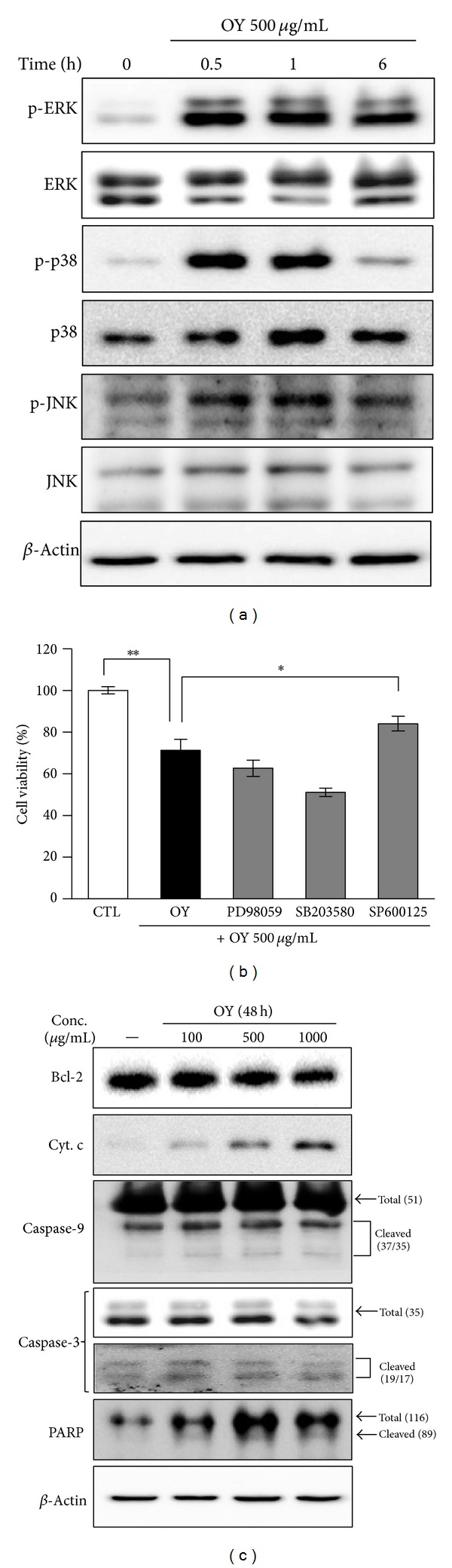
Identification of the relationship between MAPK activation and autophagy induction by treatment of OY in HCT116 cells. (a) HCT116 cells were treated with 500 *μ*g/mL OY for 0.5, 1, and 6 h. Activation of MAPKs was determined through the detection of phosphorylated protein levels by Western blot analysis. *β*-Actin was used as a loading control. (b) Cells were pretreated with PD98059 (10 *μ*M), SB203580 (10 *μ*M), or SP60125 (10 *μ*M) for 30 min and added OY at concentration of 500 *μ*g/mL OY, and further incubated for 48 h. Cell viability was determined using MTT assay. Values indicate the mean ± SD. *P < 0.05 and **P < 0.01 versus untreated and treated cells with OY only, respectively. (c) After treatment with OY (100, 500, or 1000 *μ*g/mL) for 48 h, cell lysates were prepared and Western blot analysis was performed against Bcl-2, Cyt. c, caspase-3, -9, and PARP. *β*-Actin was used as a loading control.

To investigate the relationship between activation of MAPK and induction of autophagy by OY, we analyzed the level of total and phosphorylated MAPKs by Western blot analysis after OY treatment. As shown in Figure 4(a), the levels of p-ERK, p-38, and p-JNK were significantly upregulated by OY. p-ERK and p-JNK especially were increased after 30 min of OY treatment and its activity remained for 6 h, which is the same point of LC3-II induction as shown in Figure 3(a). To confirm that the activation of MAPK was required for autophagy induction and subsequent cell death, the cells were pre-treated with specific inhibitors against ERK, p38, and JNK, such as PD98059, SB203580, and SP600125, respectively. When with cells were treated with 500 *μ*g/mL OY with each specific inhibitor for 48 h, we found that only SP600125 significantly decreased the cell viability induced by OY. In contrast, neither PD98059 nor SB203580 blocked the effect of OY (Figure 4(b)). Taken together, these results indicate that MAPK signals are involved in OY-induced autophagy at the early stage and the anti-proliferative effect of OY on HCT116 cells is closely related with JNK activation. To further determine whether the anti-proliferative effect of OY was related to apoptosis, the cells were treated with the indicated concentrations of OY for 48 h and the level of apoptosis-related proteins as well as caspases activation was examined by Western blot analysis. As shown in Figure 4(c), the level of Cyt. c released from mitochondria to cytosol was increased and the antiapoptotic protein Bcl-2 was decreased by the treatment with 500 *μ*g/mL and 1000 *μ*g/mL OY in HCT116 cells. However, the cleavages of caspases-3, -9, and PARP, markers of apoptosis, was weakly appeared with treatment of OY. Based on these findings, we suggest that activation of caspases did not strongly affect the cancer cell death induced by OY in HCT116 cells. 

## 4. Discussion

In the present study, we first investigated that OY has anti-cancer properties in human colon cancer cells and it is caused by the induction of autophagy. After the treatment with OY on HCT116 human colon cancer cells, we observed that the accumulation of cytoplasmic vacuoles and its morphological changes had a critical effect on cell proliferation. OY is composed of twelve herbs and some of the constituent herbs have been reported to have anti-cancer effect [18–21]. The remaining water phase from the methanol extract of Ephedra Herb especially has antitumor activity against mouse melanoma cells [24]. Our results also showed that water extract of Ephedra Herb (1000 *μ*g/mL) strongly inhibited the viability of HCT116 cells and its effect was about 3 times higher than that of OY in HCT116 cells. Further, Ephedra Herb exhibited cytotoxicity on normal cells, about 30% as compared with untreated cells. However, even though the water extract of Ephedra Herb had an obvious anti-cancer effect against HCT116 cells, its effect was not related with autophagy induction such as vacuoles formation in cells. 

Autophagy helps cells to survive under conditions of starvation or growth factor withdrawal, but excessive autophagy could trigger cell death [1]. Autophagy generates vacuoles called autophagosome in cytosol, which is estimated by detecting the level of LC3-II [22]. LC3 consists of two forms, LC3-I and its cleavage form, LC3-II. The LC3-II/-I ratio directly correlates with the formation of autophagosomes [23]. Our results showed that OY remarkably elevated LC3-II level in a dose- and time-dependent manner. Based on these results, we used 3-MA, an inhibitor of autophagy to, check whether OY triggers autophagic cell death [25]. As a result, 3-MA reduced autophagosome formation by OY in HCT116 cells (Figure 3(b)). Further, when we cotreated OY and 3-MA, LC3-II level was decreased compared with that of OY treatment alone (Figure 3(c)). Interestingly, though 3-MA blocked the formation of autophagosome, 3-MA did not recover the cell proliferation inhibited by OY. This result supposes that 3-MA may induce cell death as a phosphoinositide 3-kinase (PI3K) inhibitor at a later step in HCT116 cells. It has been reported that a group of PI3K inhibitors including 3-MA, wortmannin, and LY294002 works as autophagy inhibitors [26]. Because of the inhibition of PI3K signals, especially suppression of essential proteins for induction of autophagy like mTOR, 3-MA inhibits LC3-II induction in the early stage (about 6 h) and it induces the accumulation of autophagic markers in the late stage (6 h later) [27]. Since 3-MA treatment efficiently blocked the formation of autophagosomes and increase of LC3-II level, our study suggests that autophagy effect of OY may fully affect the cancer cell viability though 3-MA did not completely rescue the cell viability. To further clarify the role of MAPK activation in autophagy induced by OY, we carried out Western blot analysis and inhibitor study. Western blot analysis proposed possible mechanisms involved in the cellular action of OY *via* regulating MAPK signals. MAPKs, including p38, JNK, and ERK, are being activated by extracellular signals, which control cell proliferation, differentiation, cell death, and autophagy [28–31]. Especially, MAPKs take an important role in autophagy, which is connected to cell death or survival [32]. When we investigated cross-talk between MAPK signaling pathway and autophagy induced by OY using specific inhibitors, such as PD98059, SB203580, or SP600125, we found that OY-induced cell death mainly depends on JNK activation. 

When we checked the apoptotic effect of OY using Western blot analysis, the decrease in Bcl-2 and release of Cyt. c were caused by OY, whereas caspase activation was not. Some previous reports demonstrated that downregulation of Bcl-2 triggers autophagic cell death without involvement of mitochondrial signaling rather than apoptosis in human leukemic cells [33]. Other reports also demonstrated that Bcl-2 interacts with beclin-1, a critical marker of autophagy, and the overexpression of Bcl-2 inhibits autophagy induction in leukemic cells [34]. On the basis of these reports, we suppose that Bcl-2 level reduced by OY might be involved in autophagy induction in HCT116 cells. Since we could not find the effect of OY on beclin-1 in this study, we are going to investigate the detailed mechanism of autophagy induced by OY in other cancer cells.

## 5. Conclusion

We found that OY causes the cell death of human colon cancer cells without toxicity on normal cells and its anti-cancer effect comes from autophagy induction. We also confirmed that OY upregulates the activation of MAPK cascades, which are involved in autophagy induction; interestingly, the significant activation of JNK is related to anti-proliferative effect in HCT116 cells. Further, our study elucidated that OY causes autophagy-mediated cell death with the induction of weak apoptotic effect. In conclusion, we suggest that OY induces autophagic cell death through activating JNK signals in cancer cells; therefore, OY might have a potential to be developed as an herbal anti-cancer remedy.

## Figures and Tables

**Table 1:** Composition of oyaksungisan (OY) prescription.

Scientific name	Botanical name	Amounts used (g)
*Cnidium officinale* Makino	Cnidii Rhizoma	4
*Angelica dahurica *	Angelica Dahurica Root	4
*Glycyrrhiza glabra* Fisch	Glycyrrhizae Radix et Rhizoma	1.2
*Citrus unshiu* Markovich	Citrus Unshiu Peel	6
* Bombysis Corpus*	Batryticatus Bombyx	4
*Platycodon grandiflorum *	Platycodon Root	4
*Citrus aurantium* L.	Aurantii Fructus Immaturus	4
*Ephedra sinica* Stapf	Ephedra Herb	6
*Zingiber officinale* Rosc.	Zingiberis Rhizoma	2
*Zingiber officinale* Rosc.	Zingiberis Rhizoma Crudus	1.49
*Lindera aggregata *	Lindera Root	6
*Zizphus jujuba* Miller var. *inermis* Rehder	Zizyphi Fructus	2

	Total amounts	44.69

**Table 2:** HPLC chromatogram conditions.

Time (min)	Solvent	
	A^a^ (%)	B^b^ (%)
0	80	20
10	80	20
15	65	35
30	50	50
40	35	65
50	35	65
60	30	70

^a^0.1% trifluoroacetic acid water.

^b^Methanol.
